# Magnetic Nanoparticle-Based Ligand Replacement Strategy for Chemical Luminescence Determination of Cholesterol

**DOI:** 10.3389/fchem.2020.601636

**Published:** 2020-11-11

**Authors:** Yalan Wu, Danfeng Peng, Zhiwen Qi, Jing Zhao, Wenyi Huang, Ying Zhang, Changhui Liu, Tao Deng, Fang Liu

**Affiliations:** ^1^Institute of Tropical Medicine and Artemisinin Research Center, Guangzhou University of Chinese Medicine, Guangzhou, China; ^2^Institute of Chemical Industry of Forest Products, Chinese Academy of Forestry, Nanjing, China; ^3^School of Pharmaceutical Sciences, Guangzhou University of Chinese Medicine, Guangzhou, China

**Keywords:** cholesterol detection, host-guest interaction, chemical luminescence assay, Hemin, smartphone

## Abstract

Determination of serum cholesterol (Chol) is important for disease diagnosis, and has attracted great attention during the last few decades. Herein, a new magnetic nanoparticle-based ligand replacement strategy has been presented for chemical luminescence detection of Chol. The detection depends on ligand replacement from ferrocene (Fc) to Chol through a β-cyclodextrin (β-CD)–based host–guest interaction, which releases Fc–Hemin as a catalyst for the luminol/hydrogen peroxide chemical luminescence system. More importantly, the luminescence signal can be captured by the camera of a smartphone, thus realizing Chol detection with less instrument dependency. The limit of detection of this method is calculated to be 0.18 μM, which is comparable to some of the developed methods. Moreover, this method has been used successfully to quantify Chol from serum samples with a simple extraction process.

## Introduction

Cholesterol (Chol) is a type of biological lipid, which could be generated by the liver or from the daily intake of fat. It is essential for the formation of cell membranes, vitamin D, and several steroid hormones such as glucocorticoids, estrogen, and progesterone that are vital signal molecules for mammalians (Tabas, 2002). Chol can exist in blood as free molecules or be carried with lipoproteins. Normally, the total Chol in blood should be <200 mg/dL (5.17 mM), whereas >240 mg/dL (6.21 mM) is defined as the high level that has often been linked to many diseases such as nephrosis, diabetes mellitus, myxedema, heart, and vascular diseases (Sekretaryova et al., 2014; Liang et al., 2017). Therefore, the determination of Chol level in blood has gained increasing importance in clinical analysis and diagnosis.

To date, multiple analyzing assays have been developed for blood Chol, which could be divided into two types, the enzyme-based methods and non–enzyme-based methods. In most enzyme-based systems, Chol oxidase (ChOx) is applied to oxidize free Chol to cholest-4-en-3-one. Hydrogen peroxide (H_2_O_2_) is generated as a side product of the enzymatic reaction, which can be easily detected using colorimetric, fluorometric, or chemical luminescent probes of H_2_O_2_ (Li et al., 2019; Narwal et al., 2019). Following the similar concepts, many subtypes of ChOx-based detection assays have been developed. In the last decades, such H_2_O_2_ generation reactions have been integrated with nanotechniques that have led to several much advanced nanosensors (Huan et al., 2015; Huang et al., 2018; Ma et al., 2018; Wang et al., 2018; Thakur et al., 2019; Xu et al., 2019; Zhao et al., 2019). Nevertheless, integral drawbacks such as high cost for enzyme manipulation and unexpected denaturation of enzymes during storage have limited their real applications. Nonenzymatic platform is thus desirable; however, only a few examples have been reported by far. Among all, the nanoplatform-based competitive host–guest chemistry has attracted the greatest interest, where β-cyclodextrin (β-CD) is often used as the host acceptor for Chol due to the strong interaction between them (Crini, 2014; Ma and Zhao, 2015; Wu et al., 2015; Lu et al., 2019). Recently, Mondal and Jana (2012) have developed a fluorescence assay by using competitive host–guest interaction between rhodamine 6G (R6G) and Chol with graphene-bound β-CD. The fluorescence from R6G is greatly quenched by graphene, which can be recovered upon the addition of Chol, thus making quantitative analysis approachable (Mondal and Jana, 2012). A similar design has been adapted to a β-CD functionalized Fe_3_O_4_-polydopamine (Fe_3_O_4_@PDA) nanosystem, in which Chol competed with rhodamine B in binding with β-CD, thus leading to fluorescence increase (Li et al., 2018). Except for fluorescence assays, competitive host–guest interaction strategy has also been used for electrochemical systems by simply replacing the fluorescence dyes with redox indicators. Methylene blue and ferrocene are such redox indicators, which have been applied for Chol electrochemical detection by several research groups, individually (Agnihotri et al., 2015; Yang et al., 2015b, 2016; Ganganboina and Doong, 2018). We realized that the reactions emitting chemical luminescence have been used extensively for convenient and sensitive detection of many biological molecules. Combination of host–guest interaction with a chemical luminescent platform would be a promising way for Chol detection, however, such systems are still deficient.

**Scheme 1:**
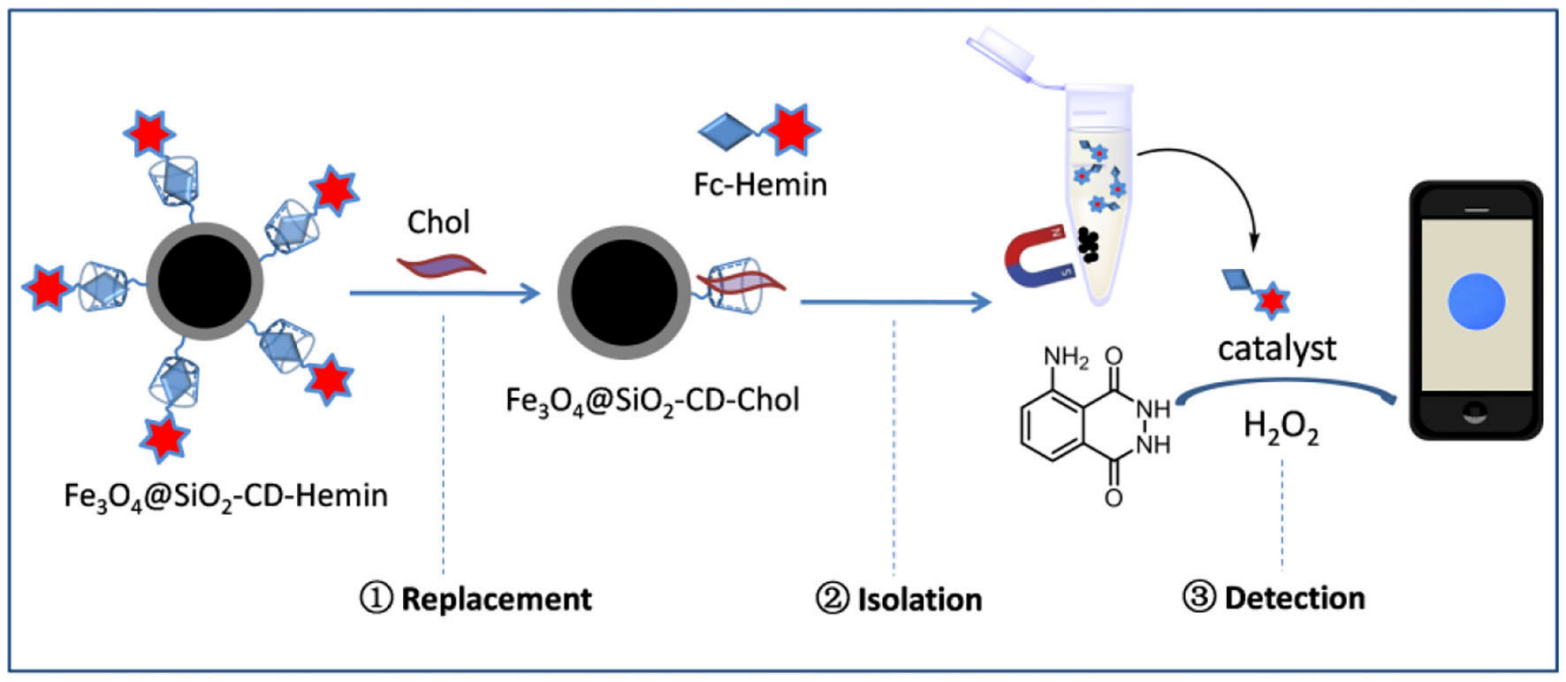
Schematic figure shows Chol detection using magnetic nanoparticle-based ligand replacement strategy.

Herein, a competitive host–guest interaction assay has been presented for chemical luminescence detection of Chol (Scheme 1). The key process of Chol detection lies on the use of a functional Fe_3_O_4_@SiO_2_ nanoparticle carrying β-CD on the surface. Hemin, a catalyst for luminol/H_2_O_2_ reaction, was linked with ferrocene to form Fc–Hemin and then bound to β-CD through host–guest interaction to form Fe_3_O_4_@SiO_2_-CD-Hemin. Upon incubation with Chol, competitive interaction occurs between Chol and Fc–Hemin, which leads to the release of free catalyst to the solution. The nanoparticles are easy to be attracted to the bottom of a testing tube; the supernatant with Hemin was then used for chemical luminescence quantification through catalyzing luminol/H_2_O_2_ reaction. More interestingly, the signal could be easily captured by the camera of a smartphone, which makes the detection less equipment dependent.

## Experimental

### Materials and Methods

Chemicals were purchased from commercial sources; all reagents were AR grade and used without further purification unless otherwise indicated. The total Chol quantification kit was purchased from Solarbio Life Science (Beijing, China), cat. no. is BC1980. The distilled deionized water from a Milli-Q Plus System was used throughout the experiments. UV-vis absorption spectra were recorded on a spectrophotometer TU-1900 (Persee, Beijing). ESI-MS was recorded by an Agilent 6420 LC/MS instrument. ^1^H and ^13^C NMR spectra were recorded on AVANCE III HD 400-MHz digital NMR spectrometer (Switzerland). Data were reported as follows: chemical shift, multiplicity (s = singlet, d = doublet, and m = multiplet), coupling constant (*J* values) in Hz, and integration. TEM images have been taken using JEM 2100F, and dynamic light scattering (DLS) analysis has been conducted on Zetasizer Nano ZS. Infrared (IR) spectra were obtained by a Nicolet iS10 Fourier transform infrared (FTIR) spectrometer. The chemical luminescence images were taken using iPhone 7 with the software Procam 7 for cameral control.

### Ligand Synthesis

**Scheme 2:**
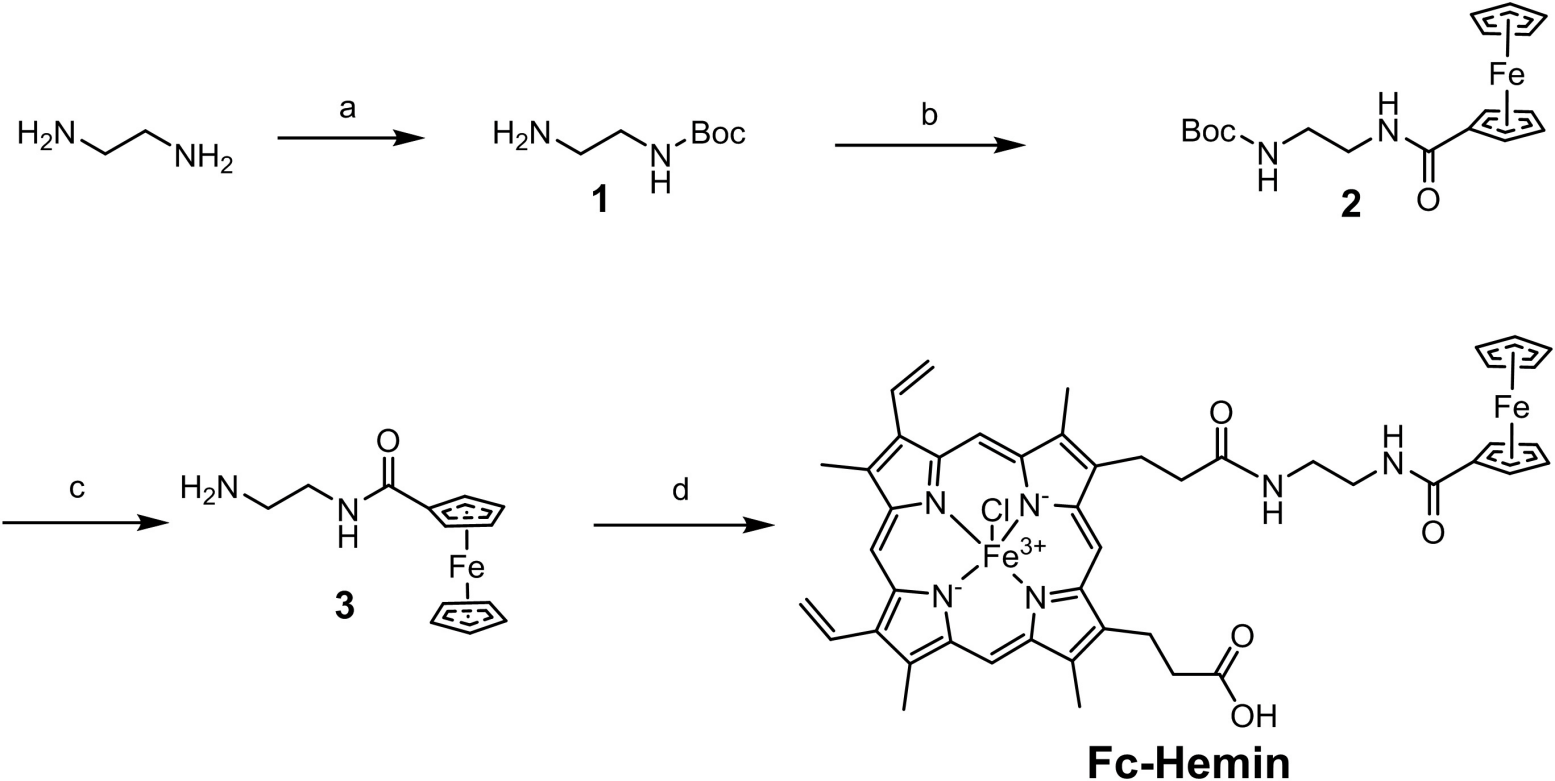
Synthetic route of Fc–Hemin. (a) (Boc)_2_O, triethylamine (TEA), ethanol, room temperature (rt); (b) 1-(3-Dimethylaminopropyl)-3-ethylcarbodiimide (EDC), 1-Hydroxybenzotriazole (HOBt), N,N-Dimethylformamide (DMF), rt; (c) Trifluoroacetic acid (TFA), Dichloromethane (DCM), rt; (d) EDC, HOBt, DMF, rt.

The synthesis of compound **1** was conducted by following a reported method (Liu et al., 2012). Ferrocene carboxylic acid (Fc-COOH) was then linked with **1** to form compound **2** through EDC/HOBt coupling. After removal of Boc-protection, compound **3** was obtained as a yellow solid. Please refer to the Supplementary Material for the synthetic details of **1** to **3**. The TFA salt of compound **3** (77 mg, 0.2 mmol), Hemin (156.3 mg, 0.24 mmol), and HOBt (32.4 mg, 0.24 mmol) were dissolved in 3 mL DMF; TEA (8 μL, 0.3 mmol) was added. EDC (0.3 mmol, 40.5 mg) was then added at 0°C. The reaction was performed at rt for 3 h (Scheme 2). An acetic acid aqueous solution (2 M) was poured into the reaction to form black precipitates, which was then collected by centrifugation. The crude product was purified by silica gel column chromatography with MeOH/DCM 1:10 as eluent, to obtain Fc–Hemin as black solid (45 mg, yield 25%). ESI (*m/z*) calcd. for [C47H46ClFe2N6O4^+^]-Cl: 870.22, found: 870.21. It failed to obtain the NMR spectra of Fc–Hemin since its paramagnetic property. This compound was further characterized by UV-vis absorption spectra and FTIR spectra with comparison to Fc-COOH and Hemin (see in the Supplementary Material).

### Fe_3_O_4_@SiO_2_-CD Preparation

Monodisperse Fe_3_O_4_ nanoparticles were prepared by thermal decomposition of an iron (III)–oleate precursor according to a well-developed method (Park et al., 2004). The final Fe_3_O_4_ nanoparticle was dispersed in hexane, and a drop of the solution was then placed onto a copper grid for TEM imaging. To prepare Fe_3_O_4_@SiO_2_ core-shell structure with amine groups, 10 mg of synthesized Fe_3_O_4_ nanoparticle was dispersed in the solvent mixture containing 60 mL cyclohexane, 9.6 mL Triton-X100, 4 mL 1-hexanol, 1.8 mL distilled water, and 600 μL NH_3_.H_2_O. After stirring at 600 to 700 rpm for half an hour, 150 μL tetraethyl orthosilicate (TEOS) in 2 mL cyclohexane was added dropwise into the reaction mixture. Twelve hours later, 5 μL (3-aminopropyl) triethoxysilane (APTS) and 5 μL TEOS in 2 mL cyclohexane were added into the mixture. After stirring for 5 h at rt, 50 mL precooled ethanol was added into the mixture, and the pH value was adjusted to around 7.0 by 1 M HCL at 0°C. The particle pellets were obtained by centrifugation at 10,000 rpm for 8 min and washed by distilled water and ethanol, successively (Liu et al., 2014). The nanoparticle-carrying amine group was then reacted with excess amount of succinic anhydride in the presence of TEA in DMF to achieve the functionalization with carboxylic acid groups. To synthesize Fe_3_O_4_@SiO_2_-CD, 100 mg Fe_3_O_4_@SiO_2_ nanoparticle carrying -COOH was mixed with 20 mg mono 6-amino β-CD in 3 mL DMF in the presence of 10 mg HOBt; 10 mg of EDC was then added to the mixture. The reaction was performed at rt for 5 h before isolation and washing.

### Photo Collection and Image Analysis

Round plastic cells with an inner diameter of 1.3 cm were used as the containers for the luminol/H_2_O_2_ reaction. In a typical test, 200 μL luminol (2 mM) in Britton–Robinson (BR) buffer (0.08 M H_3_BO_3_, 0.08 M H_3_PO_4_, and 0.08 M CH_3_COOH, pH 12) was mixed with 100 μL phosphate-buffered saline (PBS) that potentially contains Hemin; 100 μL H_2_O_2_ solution (10 mM in distilled water) was then added. The reaction can emit blue luminescence immediately upon the addition of H_2_O_2_ in the presence of Hemin. The reaction was performed under dark for easy photo-taking using a smartphone. iPhone 7 with the camera software Procam 7 was applied to take the image. The chemical luminescence was continually recorded by the phone with 8-s exposure. The second image from each test was used for analysis by ImageJ. A figure was opened by ImageJ; the relative luminescence [relative light unit (RLU)] was then extracted by performing the operation “elliptical selection–analyze–histogram” successively. The detailed information for image analysis can be found in the Supplementary Figure 1. Three repeats were performed for each treatment. The final data were presented as the average value plus standard derivation.

### Chol Extraction From Serum

Human serum was obtained from a commercial source. To extract the lipid components, 1 mL serum was mixed with 5 mL organic solvent mixture of 1-hexane:isopropanol (vol/vol 2:1) in a 15-mL centrifugal tube. The mixture was agitated by a vortex generator for 30 s. The tube was centrifuged at 10,000 rpm for 5 min. The organic layer was collected. The aqueous phase was extracted once again with the same amount organic solvent mixture. The lipids in the organic phase were combined and condensed by removal of the organic solvent. The residues were then redissolved by 1 mL isopropanol and placed in the fridge at 4°C before use. To match the linear ranges of a commercial quantification kit and the presented method, Chol in isopropanol has to be diluted for 10 times and 1,000 times with PBS (pH 7.4) before analysis, respectively.

## Results and Discussion

### Hemin Catalyzed Luminol/H_2_O_2_ Reaction

Luminol/H_2_O_2_/horseradish peroxidase (HRP)–based chemical luminescence system has been used for various biochemical detection. Generally, the luminescence intensity from luminol/H_2_O_2_/HRP reaction without any other additive is relatively weak. That is the reason why an additional enhancer such as 4-iodophenol is usually required in real application (Yang et al., 2015a). Hemin is an iron-containing porphyrin, which has been found to have the similar catalytic function to HRP in luminol/H_2_O_2_ reaction (Luo et al., 2015; Yang et al., 2015a; Gao et al., 2017). Hemin-catalyzing luminol oxidation by H_2_O_2_ is normally performed under basic pH conditions without the requirement of enhancers. Recently, cell phone–based biosensing assay has been attracting increasing interests because of its promising simplicity in image capturing and processing (Kim et al., 2017; Qian et al., 2020; Zhao et al., 2020). In terms of this Chol detection system, whether the luminescence signal is bright enough to be captured using a smartphone is unclear.

**Figure 1:**
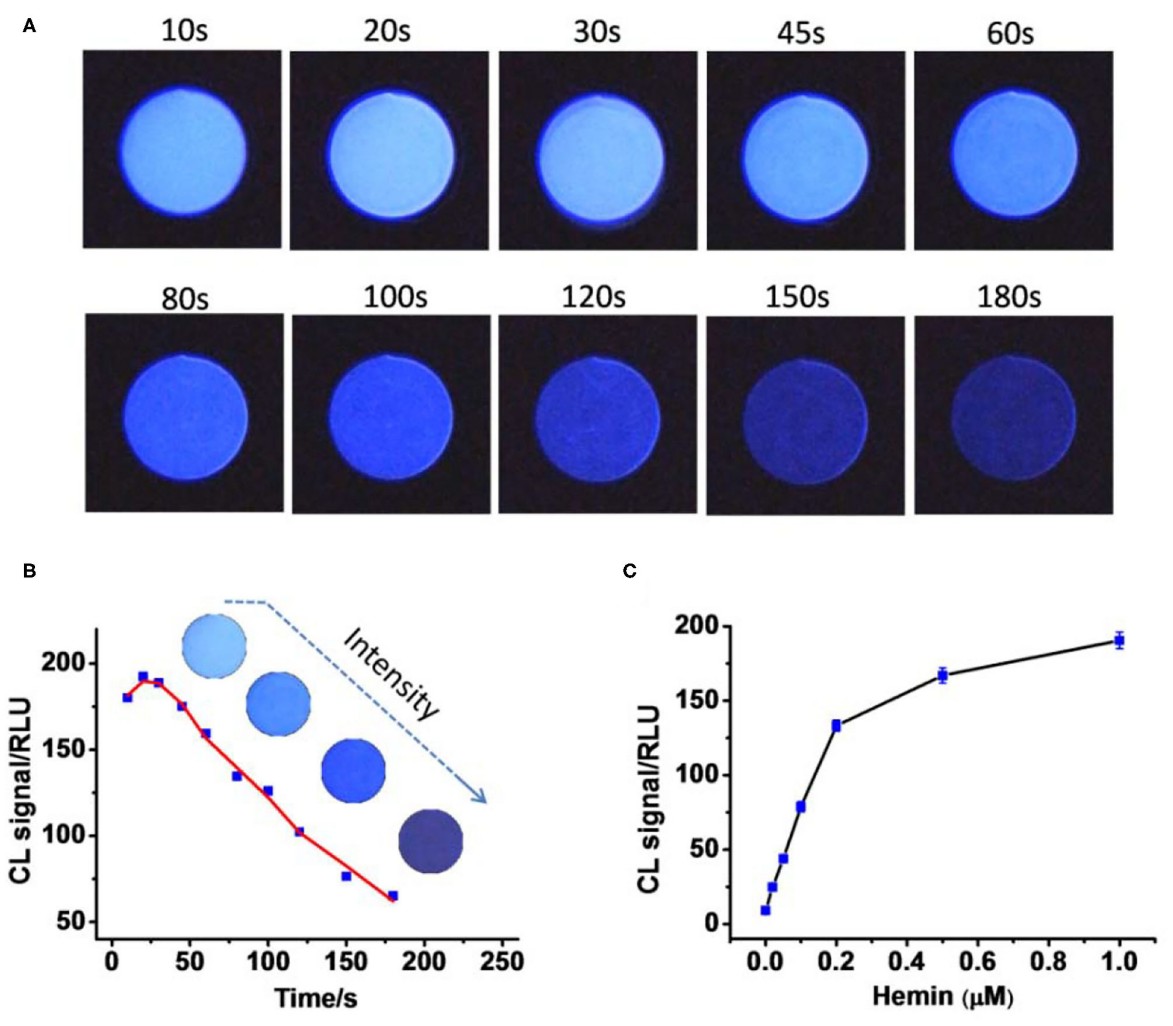
**(A)** The continual images of chemical luminescence from the reaction mixture in the presence of 1 μM. **(B)** Quantitative result shows that luminescence intensity decreases as time passes; the data were from **(A)**. **(C)** Plots of luminescence intensity against the concentration of Hemin (0, 0.02, 0.05, 0.1, 0.2, 0.5, 1 μM). Photo images were taken using a smartphone with 8-s exposure for each.

To do it, Hemin (1 μM) was mixed with luminol (2 mM) and H_2_O_2_ (10 mM) in a round plastic cell with an inner diameter of 1.3 cm. iPhone 7 was used to capture the images through the camera software Procam 7 with 8-s exposure. The images were then analyzed by the software ImageJ, where the luminescence intensity was described as RLUs. Figures 1A,B show that the light-emitting reaction is very fast. The intensity will reach its maximum values within the first 10 s followed by rapid decreasing. The reaction was then performed in the presence of Hemin at different concentrations. As indicated in Figure 1C, luminescence intensity exhibited an obvious Hemin concentration–dependent manner, thus making great potential to perform Hemin quantification by this simple way of image capturing and analyzing.

### Preparation of Fc–Hemin and Fe_3_O_4_@SiO_2_-CD Nanoparticle

Hemin was then linked with Fc-COOH through three-step organic reactions to form Fc–Hemin. The synthetic procedure and the characterization of products can be found in the Supplementary Material. Because of the paramagnetic properties of Fc–Hemin, we have failed to obtain the corresponding NMR spectra. IR spectra, UV-vis absorption spectra, and ESI-MS data are presented in the Supplementary Material. The UV-vis spectrum of Fc–Hemin in Supplementary Figure 2 shows the typical absorption properties from both Fc-COOH and Hemin, which indicates the success of the conjugation. Supplementary Figure 3 shows that most of the IR signal of Hemin and Fc-COOH overlapped in the IR spectrum of Fc–Hemin. A new peak near 1,540 cm^−1^ is found, which is attributed to amide II (N–H bending and C–N stretching) (Alver and Parlak, 2017; Balan et al., 2019). The peaks near 2,900 cm^−1^ are mostly attributed to CH groups from porphyrin structure, which can also be found from the spectrum of Hemin. The catalytic ability of the synthetic Fc–Hemin was then evaluated by comparison to free Hemin.

**Figure 2:**
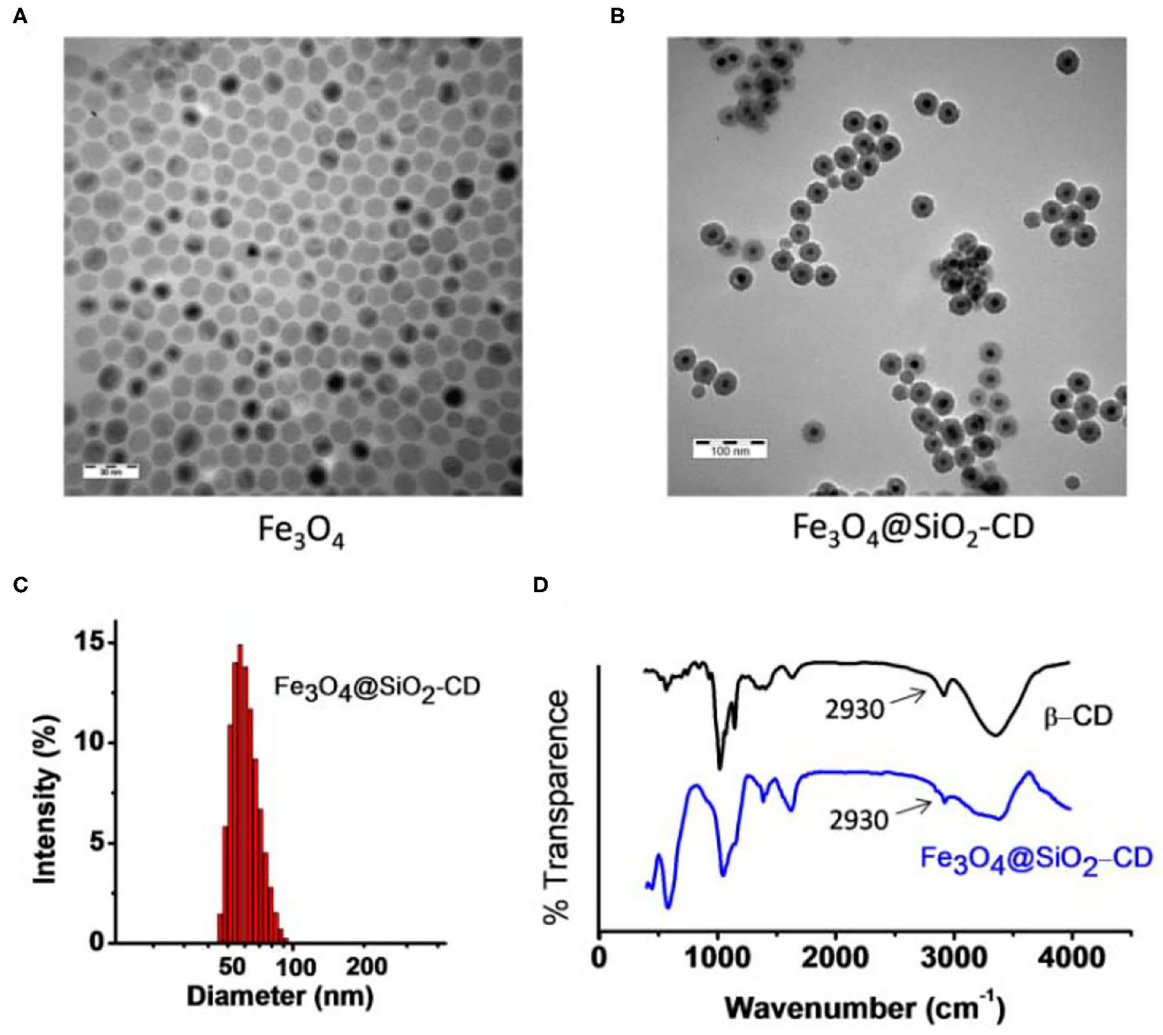
**(A)** TEM image of synthetic Fe_3_O_4_ magnetic nanoparticle. **(B)** TEM image of Fe_3_O_4_@SiO_2_-CD. **(C)** DLS result shows size distribution of Fe_3_O_4_@SiO_2_-CD nanoparticle. **(D)** FTIR spectra of Fe_3_O_4_@SiO_2_-CD and free β-CD. The arrow points to a typical signal from C-H stretching vibrations of β-CD.

The result from Supplementary Figure 4 indicates that Fc-COOH either in free form or in bound form does not affect Hemin's catalytic effect to the light-emitting reaction. Fc–Hemin exhibited comparable catalytic activity to free Hemin. Preparation of Fe_3_O_4_@SiO_2_-CD started from the synthesis of carboxylic acid functionalized Fe_3_O_4_@SiO_2_ core-shell magnetic nanoparticles following the procedure described in the *Experimental* section. Mono 6-amino β-CD was then linked to the surface of carboxylic group–carrying Fe_3_O_4_@SiO_2_ through EDC-catalyzed amide formation. TEM images show that the prepared Fe_3_O_4_ nanoparticle exhibits a relatively homogeneous size distribution with around 10- to 15-nm diameter. In addition (Figure 2A), the final Fe_3_O_4_@SiO_2_-CD nanoparticle shows a well-shaped core-shell structure and narrow size distribution from the measurement by TEM and DLS, as found in Figures 2B,C. The presence of β-CD on the nanoparticle was confirmed by FTIR spectrum (Figure 2D), in which 2,930 cm^−1^ was assigned to C–H stretching vibrations of β-CD (Kfoury et al., 2014).

### Chol Detection

To include the Fc–Hemin onto Fe_3_O_4_@SiO_2_-CD nanocarriers, both of them were mixed and incubated in PBS buffer (pH 7.4) in a plastic tube for 15 min at rt. The nanoparticle carrying Fc–Hemin was then attracted to the wall of the tube. The supernatant was then collected for the quantification of Fc–Hemin left, and the pellets were washed by fresh PBS for three times before further use (Supplementary Figure 5A). The decreased absorbance at 403 nm from the supernatant was then measured and used to estimate the amount of Fc–Hemin captured by Fe_3_O_4_@SiO_2_-CD (Supplementary Figure 5B). According to the standard calibration curve of UV-vis absorbance at 403 nm against the concentration of free Fc–Hemin in Supplementary Figure 5C, the amount of Fc–Hemin on Fe_3_O_4_@SiO_2_-CD-Hemin was estimated to be 43 μM per 1.0-mg nanoparticle.

**Figure 3:**
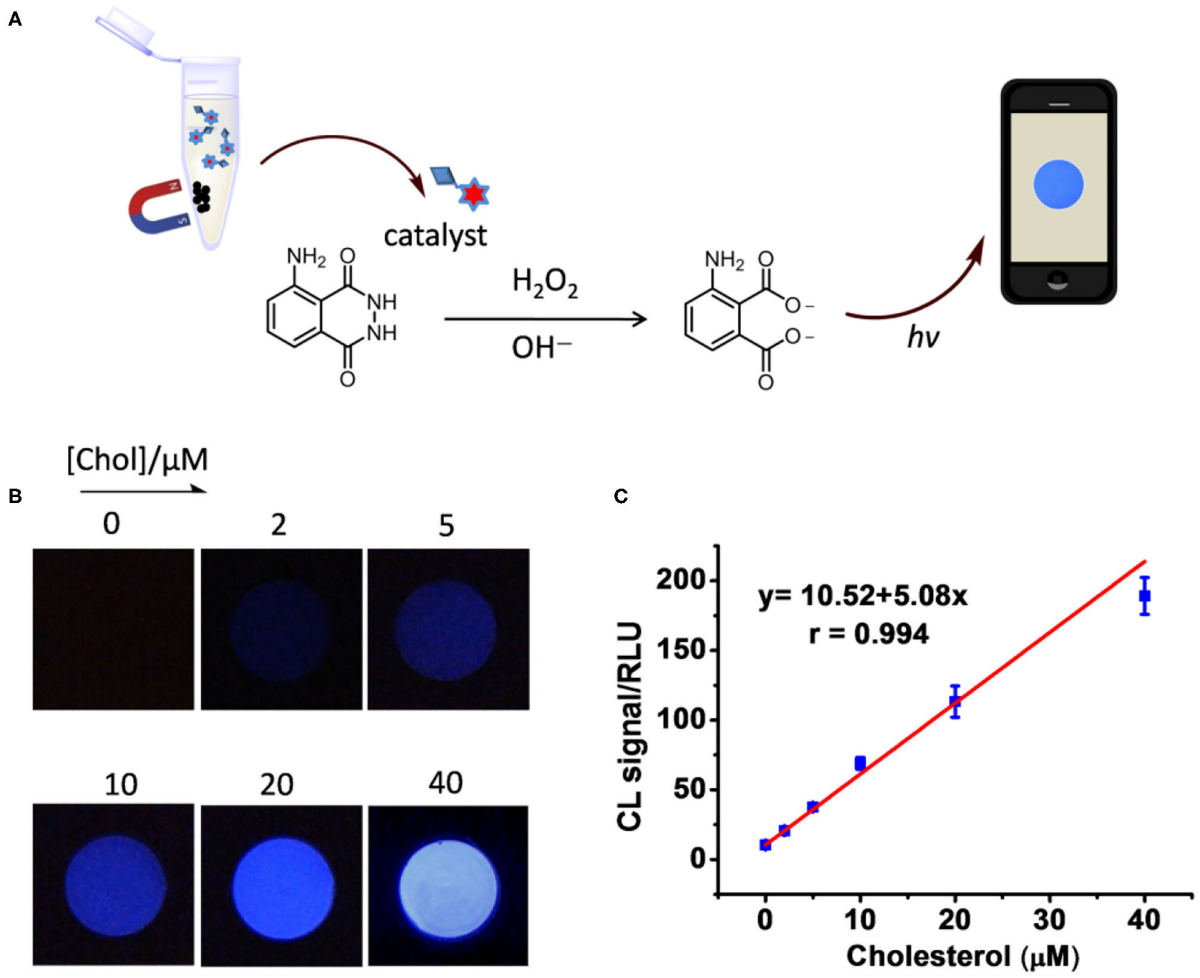
**(A)** Schematic figure shows the procedure of Chol detection. **(B)** Photo images taken using a smartphone in the detection of Chol at different concentrations (0, 2, 5, 10, 20, 40 μM). **(C)** Luminescence intensity was analyzed by ImageJ and plotted as the function of concentration.

The catalyst integrated Fe_3_O_4_@SiO_2_-CD-Hemin was then utilized for Chol detection through the smartphone-based visualizing method. To obtain a standard calibration curve, series of concentrations of Chol were incubated with 250 μg/mL Fe_3_O_4_@SiO_2_-CD-Hemin in PBS buffer for 15 min at rt. After attracting by a magnet, the supernatant was then collected for test (Figure 3A). For a typical test, 100 μL of the supernatant was mixed with 200 μL luminol (2 mM) in BR buffer (pH 12); 100 μL H_2_O_2_ (2 mM in distilled water) was then added. The chemical luminescence images were taken using the smartphone with 8-s exposure. As shown in Figure 3B, the luminescence becomes brighter as the concentration of Chol increases. The linear relationship between Chol concentration and luminescence signal (RLU) was found from 0 to 40 μM. A higher concentration >40 μM (e.g., 60 μM) will lead to the signal oversaturation under the same 8-s exposure (Supplementary Figure 6). The result indicates that ferrocene can really be replaced with Chol in β-CD host–guest systems. It has been previously found that the binding capacity between Chol and β-CD is stronger than that between Fc and β-CD (Ganganboina and Doong, 2018). Theoretic molecular docking also reveals that Chol exhibits greater potential than an Fc derivative in binding with β-CD, as indicated by the binding energy −21.86 kcal/mol for β-CD/Fc and −30.49 kcal/mol for β-CD/Chol (Supplementary Figure 7). The limit of detection was estimated to be 0.18 μM (signal-to-noise ratio = 3) according to calibration curve in Figure 3C. For comparison, the recently developed Chol detection systems on the basis of host–guest interaction have been summarized in Table 1. The sensitivity of this system is comparable to some of the reported methods based on electrochemical and fluorescence strategies. It is worth noting that this is the first example to combine a chemical luminescent system and host–guest interaction for enzyme-free detection of Chol. In addition, chemical luminescence signals are usually easier to be captured and analyzed in comparison with fluorescence or electrochemical signals, thus making the detection more convenient.

**Table 1:** The comparison of the method in this work with previously developed systems based on the same host–guest interaction.

**No.**	**Signals**	**Materials**	**Linear range**	**LOD**	**References**
1	Electrochemical	β-CD@N-GQD	0.5–100 μM	80 nM	Ganganboina and Doong, 2018
2	Electrochemical	Grp–β-CD	0–100 μM	1 μM	Agnihotri et al., 2015
3	Electrochemical	MWCNTs–β-CD/SPCE	1 nM−3 μM	0.5 nM	Nawaz et al., 2018
4	Fluorescence	β-CD–CQD	0–110.0 μM	0.7 μM	Sun et al., 2017
5	Fluorescence	β-CD–graphene-R6G	—	—	Mondal and Jana, 2012
6	Fluorescence	Fe_3_O_4_@PDA–PBA–CD	10–100 nM	4.3 nM	Li et al., 2018
7	Chemical luminescence	Fe_3_O_4_@SiO_2_-CD–Hemin	0–20 μM	0.18 μM	This work

*N-GQD, N-doped graphene quantum dots; Grp, graphene; MWCNT, multiwalled carbon nanotube; SPCE, screen printed carbon electrode; CQD, carbon quantum dots; R6G, rhodamine 6G; PDA, polydopamine; PBA, phenylboronic acid; LOD, limit of detection*.

**Figure 4:**
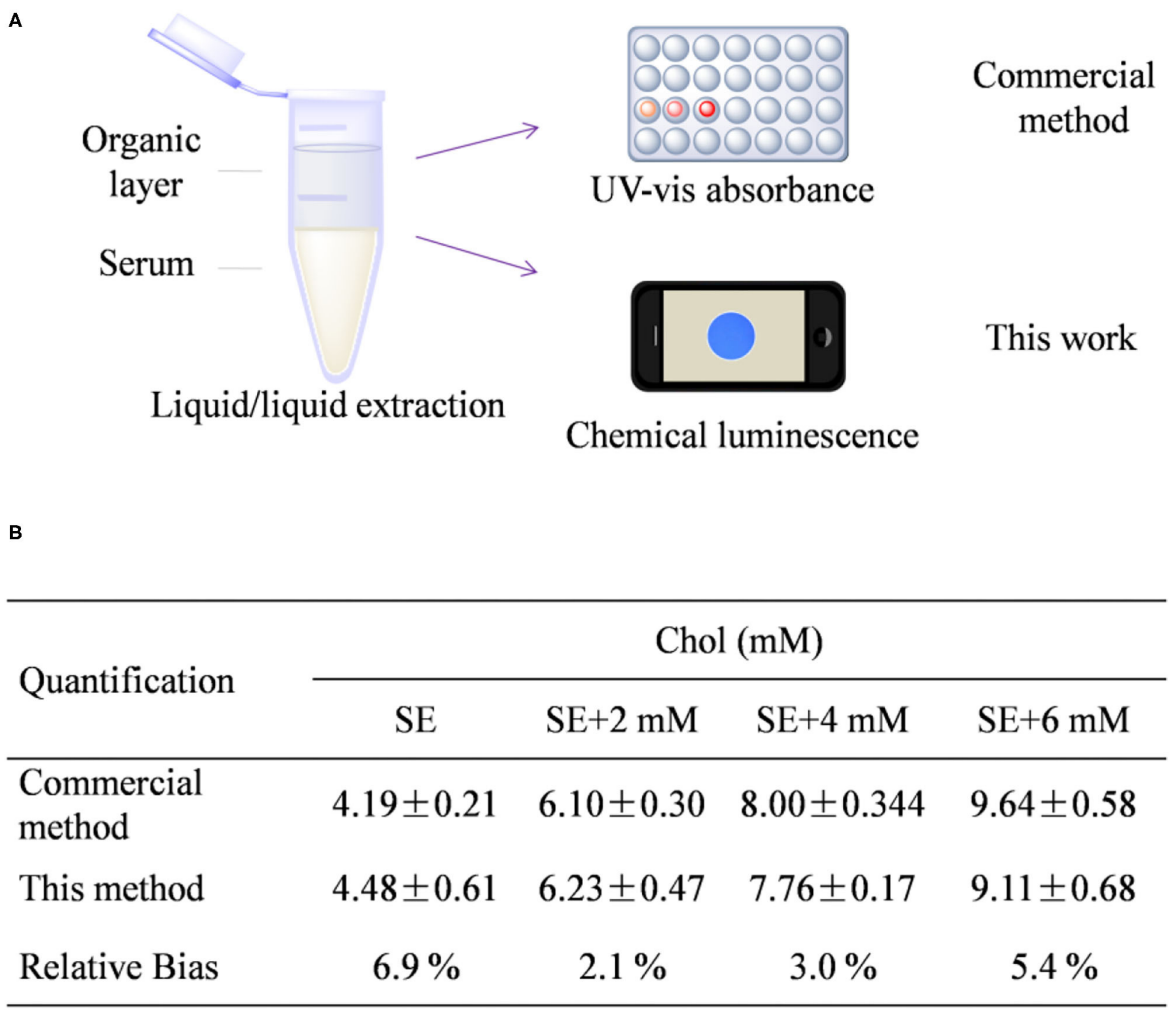
**(A)** Schematic figure shows the procedure of Chol extraction and detection. **(B)** Quantitative detection result from the presented method and a commercial kit. SE stands for human serum.

With good performance in hand, we then tried to quantify the total Chol from human serum samples by comparison to a well-recognized commercial assay. To do this, commercialized human serum was added with additional extraneous Chol of 0, 2, 4, and 6 mM, respectively. The original serum and the serum samples plus Chol additive were set as four individual samples for measurement. Before measurement, the total Chol was extracted by a liquid-to-liquid extraction method using the mixture of 1-hexane/isopropanol (vol ratio 2:1) as the organic phase (Figure 4A), following the procedure described in the *Experimental* section. This extraction method has been proven to be effective to extract >90% of total Chol from serum (Ferraz et al., 2004). At first, the concentration of Chol was quantified by a well-recognized enzyme-based colorimetric method using a commercial kit by following the protocol supplied with kit (Supplementary Figure 8). As found in Figure 4B, total Chol from the extraction of original serum was quantified to be 4.19 ± 0.2 mM, which is reasonable because the normal concentration from healthy people is usually between 3.5 and 5.17 mM (Lai et al., 2015; Yi et al., 2019). The Chol concentration from the sample was determined to be 4.48 ± 0.61 mM through the chemical luminescence method. The relative bias between these two methods was calculated to be 6.9, 2.1, 3.0, and 5.4% for the all the tested samples SE, SE + 2 mM, SE + 4 mM, and SE + 6 mM, respectively. The average relative bias is calculated to be 4.35%. In general, the relative bias <5% is desirable for a new method to compare with a standard technique (Johnson, 2008). Therefore, the result implies the great potency to use this chemical luminescence assay for Chol quantification from sera.

## Conclusion

A new functional magnetic nanoplatform has been presented for Chol quantification. The nanoparticle is prepared by functionalization of a Fe_3_O_4_@SiO_2_ with the β-CD followed by inclusion of an Fc–Hemin complex through host–guest interaction. Chol can compete with ferrocene in binding with β-CD, thus leading to the release of Fc–Hemin, an effective catalyst for the most common chemical luminescence system, luminol/H_2_O_2_. Moreover, the chemical luminescence is easy observe using the camera of a smartphone, thus making the detection more precise and less equipment-dependent. More importantly, this method has been successfully applied to quantify serum Chol with the assistance of a simple liquid extraction process. To the best of our knowledge, this is the first example to combine host–guest interaction and chemical luminescence for Chol quantification that offers a potential new alternative for practical application.

## Data Availability Statement

The original contributions presented in the study are included in the article/Supplementary Materials, further inquiries can be directed to the corresponding author/s.

## Author Contributions

TD and FL were responsible for designing the project and experiments. YW, DP, JZ, and WH have conducted the experiments. ZQ and CL were responsible for discussing and revising the manuscript. WH and YZ have conducted molecular docking. All authors contributed to the article and approved the submitted version.

## Conflict of Interest

The authors declare that the research was conducted in the absence of any commercial or financial relationships that could be construed as a potential conflict of interest.
