# Supplementary material for: Magnetic Nanoparticle-Based Ligand Replacement Strategy for Chemical Luminescence Determination of Cholesterol
Source: Front Chem. 2020 Nov 11;8:601636. doi: 10.3389/fchem.2020.601636 (PMC7693431; doi:10.3389/fchem.2020.601636)
Supplement: Supplementary file 1 [file Data_Sheet_1.docx]

**Supplementary Material**

**Magnetic Nanoparticle-based Ligand Replacement Strategy for Chemical Luminescence Determination of Cholesterol**

Yalan Wu ^1^ *^,^*^#^, Danfeng Peng ^1, #^, Zhiwen Qi ^2^, Jing Zhao ^1^, Wenyi Huang ^1^, Ying Zhang ^1^, Changhui Liu ^3^, Tao Deng ^1,^ *, Fang Liu ^1,^ *

*^1^ Institute of Tropical Medicine and Artemisinin Research Center, Guangzhou University of Chinese Medicine, Guangzhou 510405, Guangdong, PR. China.*

*^2^ Institute of Chemical Industry of Forest Products, CAF Nanjing 210042, Jiangsu, P.R. China.*

*^3^School of Pharmaceutical Sciences, Guangzhou University of Chinese Medicine, Guangzhou 510405，Guangdong, PR. China.*

**Corresponding Author：**

Tao Deng: [dengtao@gzucm.edu.cn](mailto:dengtao@gzucm.edu.cn),

Fang Liu: [fangliu@gzucm.edu.cn](mailto:fangliu@gzucm.edu.cn)

**^#^** These authors contributed equally to this work.

**1.** **Synthesis of Fc-Hemin**

Compound **1**

The synthesis and purification was conducted by following a reported method ^1^. Briefly, Ethylenediamine 2.0 mL (30 mM), triethylamine (TEA) 1.5 mL was mixed in 40 mL DCM. The reaction mixture was placed onto an ice bath, di-*tert*-butyl dicarbonate (2.18 g, 10 mM) in 10 mL was then added drop wise. The reaction was performed under rt for 4 h. The reactive mixture was then extracted by 2 M acetic acid, the aqueous phase was then adjusted to be pH over 10 by NaOH solution. EA was added for extraction again, the organic layer was combined. The product was collected after removal of solvent as a colorless oil. 3.8 g (yield 80%). ^1^H NMR (400 MHz, CDCl3): δ 5.29 (1H, br), 3.08 (2H, t, J= 4.0 Hz), 2.71 (2H, t, J = 4.0 Hz), 1.35 (9H, s).

Compound **3**

188 mg of compound **1** (1.2mmol) was dissolved in 10 mL DCM, carboxyferrocene (Fc-COOH, 1.0 mmol, 230.4 mg)，DMAP (0.05 mmol, 6.1 mg) were added into the solution. EDC (1.5 mmol, 230 mg) was added at 0 ^o^C，the reaction was kept at rt for 3 h. 15 mL water was added into the reaction, perform DCM extraction twice, the organic layer was combined and dried. The crude products were purified by silica gel column chromatography using methanol/DCM 1:50 as eluent to obtain **2** as a yellow solid (223 mg, yield 60%). Boc-protection was removed by stirring compound 2 (200 mg, 0.53 mmol) in 5 mL of 20% TFA/DCM solution for 30 min. The solvent was removed followed by the addition of 20 mL diethyl ether to obtain a yellow solid compound **3** quantitatively. ^1^H NMR (400 MHz, CDCl_3_) δ 4.83 (s, 2H), 4.46 (s, 2H), 4.24 (s, 5H), 3.60 (t, *J* = 8 Hz, 2H), 3.13(t, *J* = 8 Hz, 2H); ^13^C NMR (100 MHz, CDCl_3_) δ 177.5, 78.5, 74.8, 73.6, 72.3, 43.5, 41.0. ESI (*m/z*) calcd. for C13H17FeN2O, [M +H]^+^: 273.06, found: 273.07.

**Fc-Hemin**

Compound **3** (54 mg, 0.2 mmol), hemin (156.3 mg, 0.24 mmol) and HOBt (32.4 mg, 0.24 mmol) were dissolved in 3 mL DMF, TEA (80.3 µL, 0.3 mmol) was added. EDC (0.3 mmol, 40.5 mg) was then added at 0 ^o^C. The reaction was performed at rt for 3 h. 2 M acetic acid was pour into the reaction to form black precipitate, which was collected by centrifugation. The crude product was purified by silica gel column chromatography with methanol / DCM 1:10 as eluent, to obtain Fc-Hemin as a black solid (45 mg, yield 25%). ESI (*m/z*) calcd. for [C47H46ClFe2N6O4^+^]-Cl : 870.22, found: 870. 21.

It is failed to obtain the NMR spectra of Fc-Hemin since its paramagnetic property. This compound was further characterized by UV-vis absorption spectrum and FTIR spectra with comparison to ferrocene and hemin. Fc-Hemin may exist as the mixture of two isomers, since the two carboxylic acid groups are localized in the porphyrin asymmetrically.

**2. Characterization**


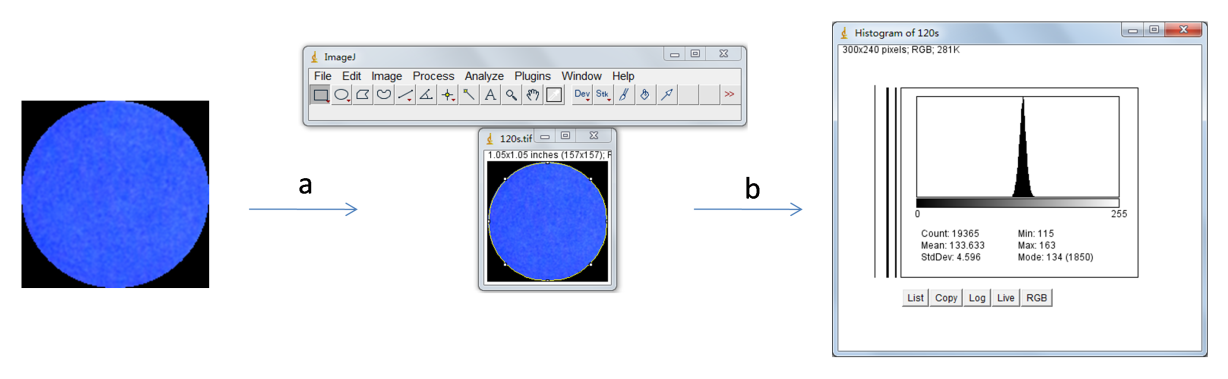


Figure S1. Image processes using Image J. a) Open the image with the software Image J, then use elliptical selection to highlight the region with luminescence; b) Click “analyze”, then click “histogram” to show the gray values. The mean values from three individual figures of each group were applied for data plotting.


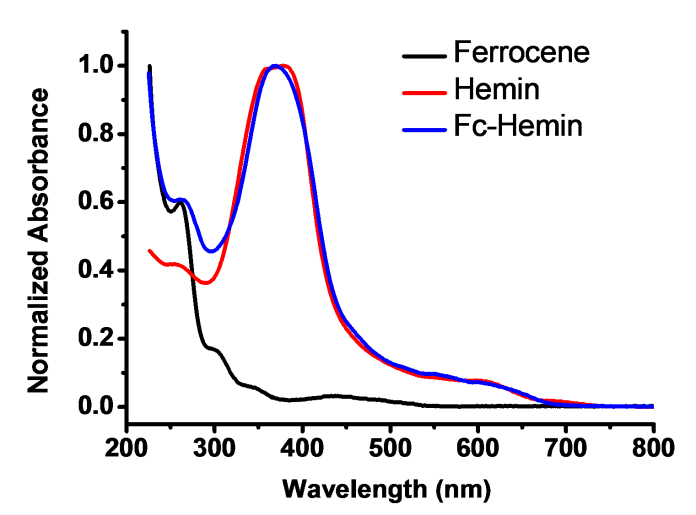


Figure S2. The UV-vis spectra of Fc-COOH, hemin and Fc-Hemin in PBS (pH 7.4). The solutions at 20 μM of each was used for measurement, the maximum abs of the spectrum was normalized to 1 for easy comparison.

Most of the IR signal of hemin and Fc-COOH overlapped in the IR spectrum of Fc-Hemin. A new peak near 1540 cm^-1^ is found, which is attributed to amide II (N–H bending and C–N ) ^2-3^. The peaks near 2900 cm^-1^ are mostly attributed to CH groups from porphyrin structure, which can also be found from the spectrum of hemin.


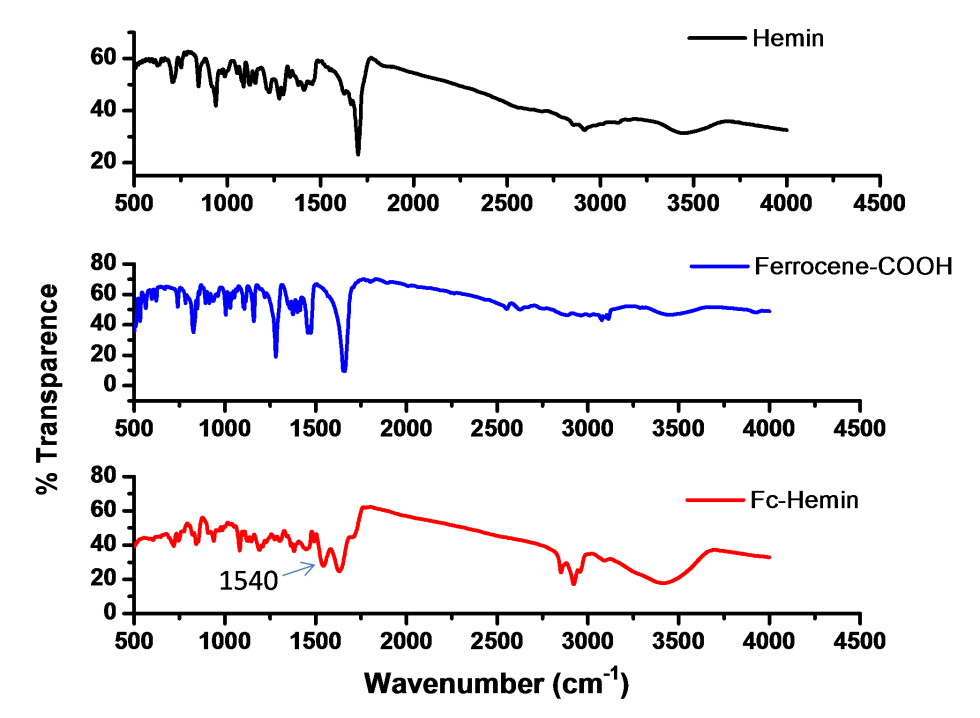


Figure S3. FTIR spectra of hemin, Fc-COOH and Fc-Hemin. KBr is used as a carrier for the sample in IR spectra.

**3. Detection**


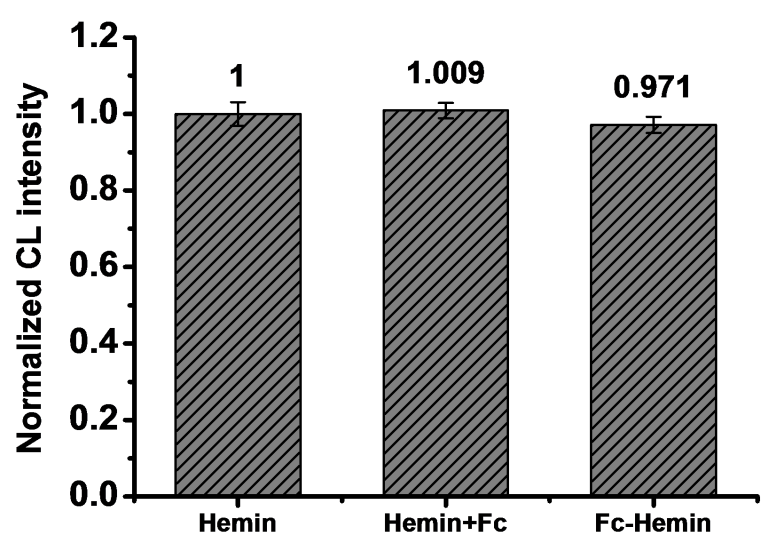


Figure S4. The CL intensity collected from the reaction catalyzed by hemin, the combination of hemin and Fc-COOH, and Fc-Hemin complex, respectively. CL intensity from the reaction with hemin was set as control and normalized to 1 for easy comparison.


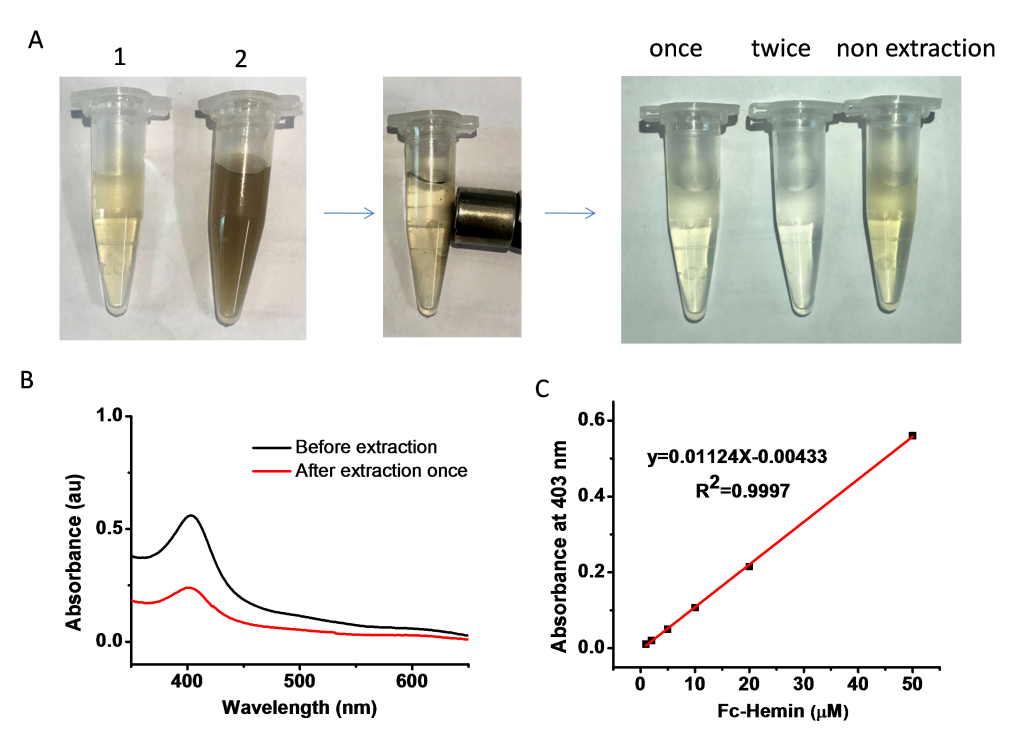


Figure S5. A) The procedure to prepare the magnetic nanoparticle carrying Fc-Hemin; B) The UV-vis spectra indicate the concentration changes of Fc-Hemin (50 µM) before and after extracting once by Fe_3_O_4_@SiO_2_-CD (500 µg/mL); C) The standard calibration curve of the absorbance at 403 nm against the concentration of Fc-Hemin.


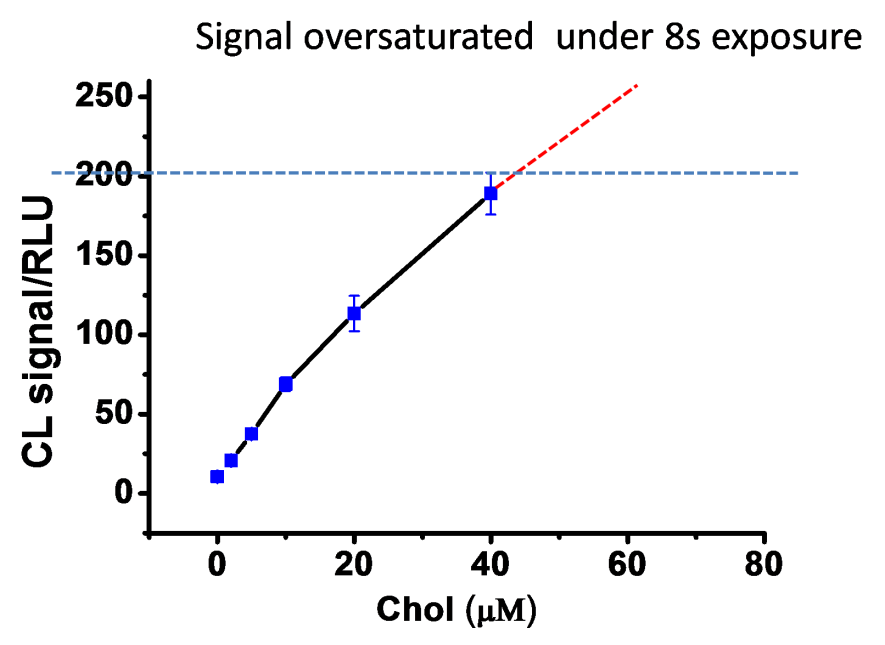


Figure S6. The signal is oversaturated when using Chol over 40 µM to perform the competitive interaction and detection.


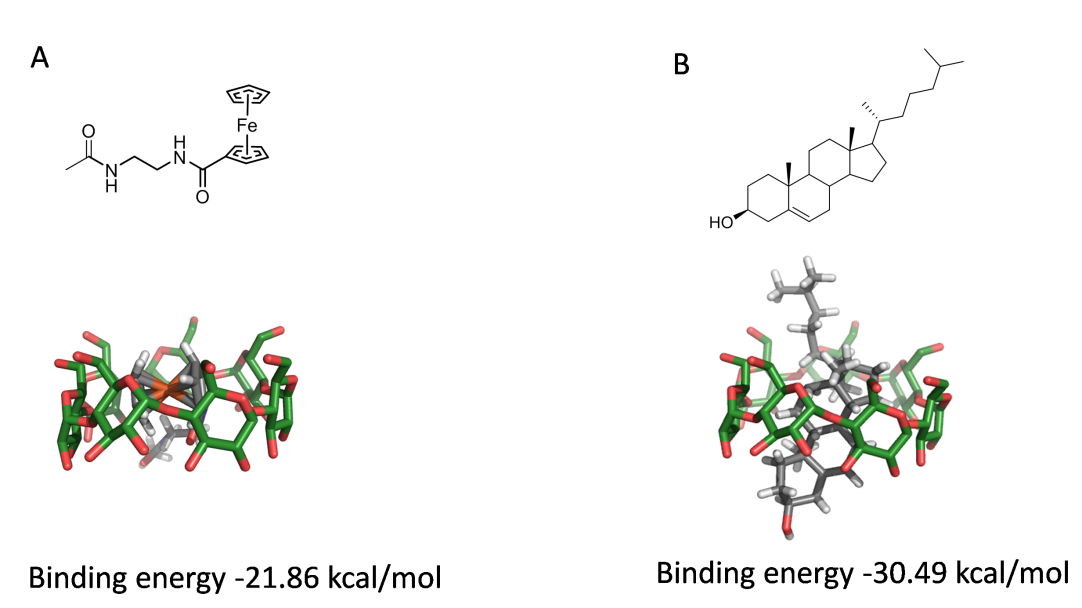


Figure S7. Molecular-docking analyzing of the binding capacity between β-CD and guest ligands N-(2-acetamidoethyl) ferrocene (A) and cholesterol (B).

The crystal structure of β-CD (PDB ID: 3cgt) was found from the open source protein data bank (PDB) https://www.rcsb.org/. The crystal structure was then opened with Sybyl-X2.1.1, the intrinsic protein ligand, surrounding water molecules and ions were removed before docking. Molecular geometry was optimized with MMFF94 force field. Multi-channel surface was set as the protomol-generation mode, the fully automatic molecular docking (Surflex) was performed. The binding energy was calculated following the function △G^o^ = -2.303*RT*Total score (where R = 8.314, T stands for kelvin temperature, herein we use 278K, total score was from docking result).


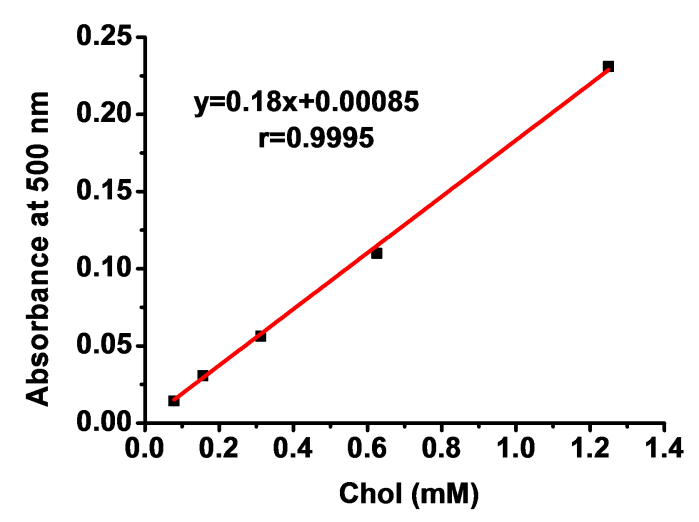


Figure S8. The standard quantification curve of the commercial Chol kit. The experiment was conducted following its standard protocol supplied with the kit.

The total Chol quantification kit was purchased from Solarbio Life science (Beijing, China), cat. No is BC1980.

**4. NMR Spectra**


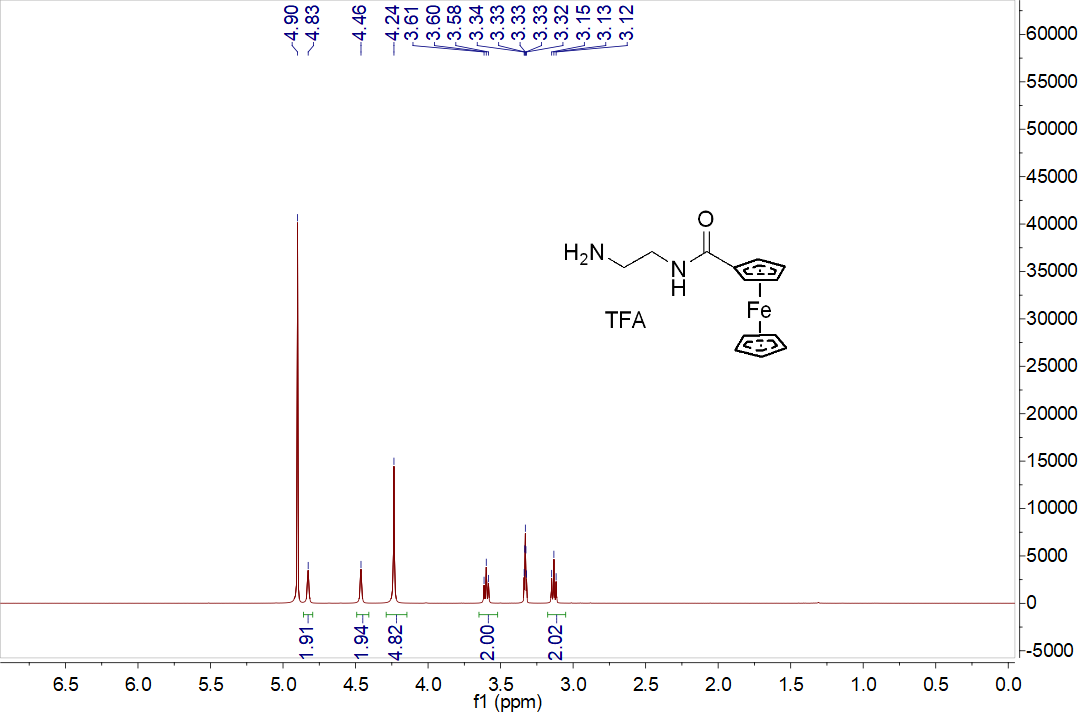


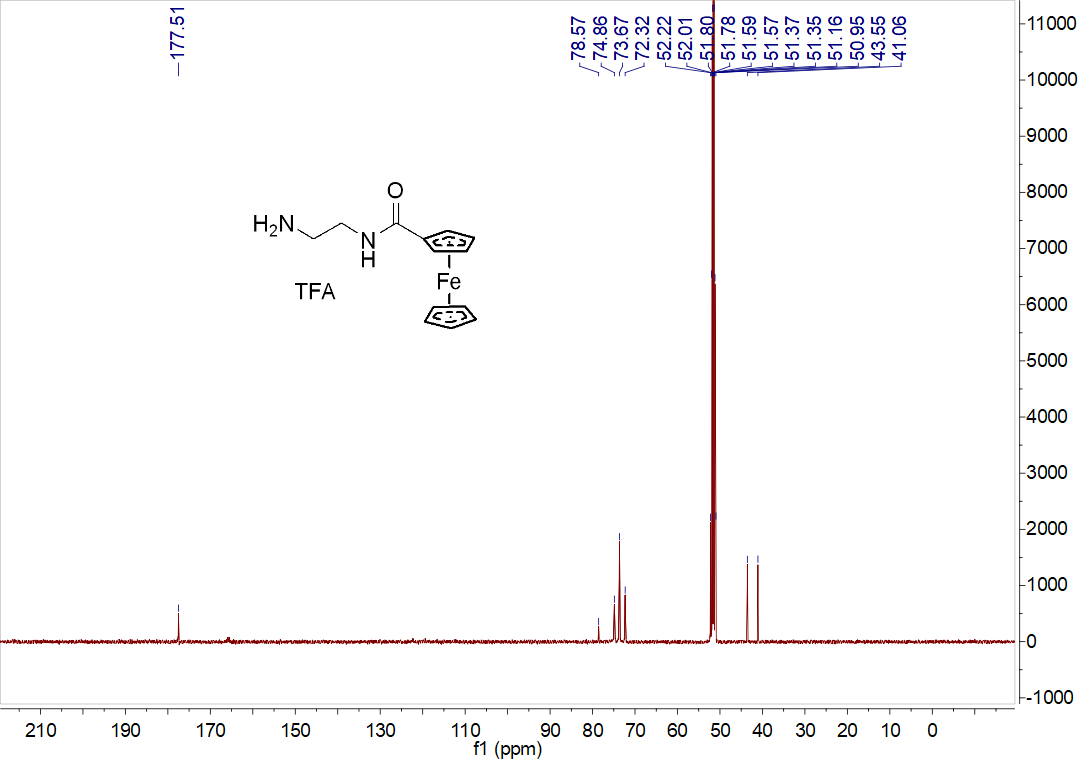


**References:**

1. Liu, F.; Soh Yan Ni, A.; Lim, Y.; Mohanram, H.; Bhattacharjya, S.; Xing, B., Lipopolysaccharide Neutralizing Peptide–Porphyrin Conjugates for Effective Photoinactivation and Intracellular Imaging of Gram-Negative Bacteria Strains. *Bioconjugate chemistry* **2012,** *23* (8), 1639-1647.

2. Alver, O.; Parlak, C., FT-IR, NMR SPECTROSCOPIC and QUANTUM MECHANICAL INVESTIGATIONS OF TWO FERROCENE DERIVATIVES. *B Chem Soc Ethiopia* **2017,** *31* (1), 63-74.

3. Balan, V.; Mihai, C. T.; Cojocaru, F. D.; Uritu, C. M.; Dodi, G.; Botezat, D.; Gardikiotis, I., Vibrational Spectroscopy Fingerprinting in Medicine: from Molecular to Clinical Practice. *Materials* **2019,** *12* (18).
